# Relevance of CCL3/CCR5 axis in oral carcinogenesis

**DOI:** 10.18632/oncotarget.16882

**Published:** 2017-04-06

**Authors:** Janine Mayra da Silva, Tálita Pollyanna Moreira dos Santos, Lays Martin Sobral, Celso Martins Queiroz-Junior, Milene Alvarenga Rachid, Amanda E.I. Proudfoot, Gustavo Pompermaier Garlet, Aline Carvalho Batista, Mauro Martins Teixeira, Andréia Machado Leopoldino, Remo Castro Russo, Tarcília Aparecida Silva

**Affiliations:** ^1^ Department of Oral Surgery and Pathology, School of Dentistry, Universidade Federal de Minas Gerais, Belo Horizonte, Minas Gerais, Brazil; ^2^ Department of Morphology, Institute of Biological Sciences, Universidade Federal de Minas Gerais, Belo Horizonte, Minas Gerais, Brazil; ^3^ Department of Clinical Analysis, Toxicology and Food Sciences, School of Pharmaceutical Sciences of Ribeirão Preto, Universidade de São Paulo, São Paulo, Bauru, Brazil; ^4^ Department of General Pathology, Institute of Biological Sciences, Universidade Federal de Minas Gerais, Belo Horizonte, Minas Gerais, Brazil; ^5^ Merck Serono Geneva Research Centre, Geneva, Switzerland; ^6^ Department of Biological Sciences, School of Dentistry, Universidade de São Paulo, SE3;o Paulo, Bauru, Brazil; ^7^ Department of Stomatology, School of Dentistry, Universidade Federal de Goiás, Goiânia, Goiás, Brazil; ^8^ Laboratory of Immunopharmacology, Department of Biochemistry and Immunology, Institute of Biological Sciences, Universidade Federal de Minas Gerais, Belo Horizonte, Minas Gerais, Brazil; ^9^ Laboratory of Pulmonary Immunology and Mechanics, Department of Physiology and Biophysics, Institute of Biological Sciences, Universidade Federal de Minas Gerais, Belo Horizonte, Minas Gerais, Brazil

**Keywords:** chemokines, CCL3, CCR1, CCR5, OSCC

## Abstract

The chemokine CCL3 is a chemotactic cytokine crucial for inflammatory cell recruitment in homeostatic and pathological conditions. CCL3 might stimulate cancer progression by promoting leukocyte accumulation, angiogenesis and tumour growth. The expression of CCL3 and its receptors CCR1 and CCR5 was demonstrated in oral squamous cell carcinoma (OSCC), but their role was not defined. Here, the functions of CCL3 were assessed using a model of chemically induced tongue carcinogenesis with 4-nitroquinoline-1-oxide (4NQO). Lineages of OSCC were used to analyse the effects of CCL3 *in vitro*. The 4NQO-induced lesions exhibited increased expression of CCL3, CCR1 and CCR5. CCL3^-/-^ and CCR5^-/-^ mice presented reduced incidence of tongue tumours compared to wild-type (WT) and CCR1^-/-^ mice. Consistently, attenuated cytomorphological atypia and reduced cell proliferation were observed in lesions of CCL3^-/-^ and CCR5^-/-^ mice. OSCC from CCL3^-/-^ mice exhibited lower infiltration of eosinophils and reduced expression of *Egf, Fgf1, Tgf-β1, Vegfa, Vegfb, Itga-4, Vtn, Mmp-1a, Mmp-2* and *Mmp-9* than WT mice. *In vitro*, CCL3 induced invasion and production of CCL5, IL-6, MMP -2, -8, -9. Blockage of CCL3 *in vitro* using α-CCL3 or Evasin-1 (a CCL3-binding protein) impaired tumour cell invasion. In conclusion, CCL3/CCR5 axis has pro-tumourigenic effects in oral carcinogenesis. The induction of inflammatory and angiogenic pathways and eosinophils recruitment appear to be the underlying mechanism explaining these effects. These data reveal potential protective effects of CCL3 blockade in oral cancer.

## INTRODUCTION

Oral squamous cell carcinoma (OSCC) is the most common malignancy of the oral cavity worldwide [1, 2]. OSCC is considered highly infiltrative, locally aggressive and frequently metastasizes to the cervical lymph nodes [1, 3].

Tumour behaviour depends on the malignant cell capabilities and the tumour microenvironment, which directly stimulates tumour growth, invasion and metastasis [4]. The tumour cells cooperation with their supporting stroma is based on the production of angiogenic factors, integrins, proteases, cytokines and chemokines [4, 5]. Chemokines may exert anti-tumour effects via recruitment of immune effector cells to the tumour microenvironment [6–8]. However, accumulating evidence suggests that chemokines might also exert pro-tumorigenic effects in different cancers [9–12], including OSCC [13–18]. The pro-tumour activities of chemokines involve the induction of inflammatory cell trafficking, neoplastic cell motility, neovascularization and extracellular matrix remodelling [9–22].

The chemokine CCL3 (previously called macrophage inflammatory protein-1α - MIP-1α) is produced by macrophages, T cells, monocytes, fibroblasts and epithelial cells [23]. CCL3 binds to its receptors CCR1 and CCR5, which are both mainly expressed on lymphocytes, monocytes, macrophages, eosinophils, natural killer and dendritic cells [23]. A few studies have demonstrated increased expression of CCL3 in human hepatomas [19], multiple myeloma [24] and chronic lymphocytic leukaemia [25]. Functional studies demonstrated that mice deficient for CCL3 and its receptor CCR1 were significantly protected from carcinogen-induced hepatocarcinogenesis *in vivo* [20]. Hepatoma cell lines were found to produce increased levels of CCL3, which in turn stimulated these cells to produce pseudopodia and to migrate *in vitro* [19, 26]. In a model of renal cell carcinoma, a decreased incidence of metastasis was observed in mice deficient for CCL3 and CCR5 [21]. In the context of OSCC, one study previously showed the expression of CCL3 and CCR1 in tumour samples and metastatic lymph nodes and correlated it with poor cumulative survival [27]. To date, there are no functional studies regarding the effect of CCL3 in oral carcinogenesis. Herein, we employed a model of chemically induced OSCC to investigate the role of the CCL3/CCR1/CCR5 axis in oral carcinogenesis. We also used *in vitro* approaches to determine the effects of CCL3 in OSCC cells.

## RESULTS

### Expression of CCL3 and its receptors CCR1 and CCR5 in experimentally-induced SCC, neoplastic cell lines and human OSCC

**Figure 1:**
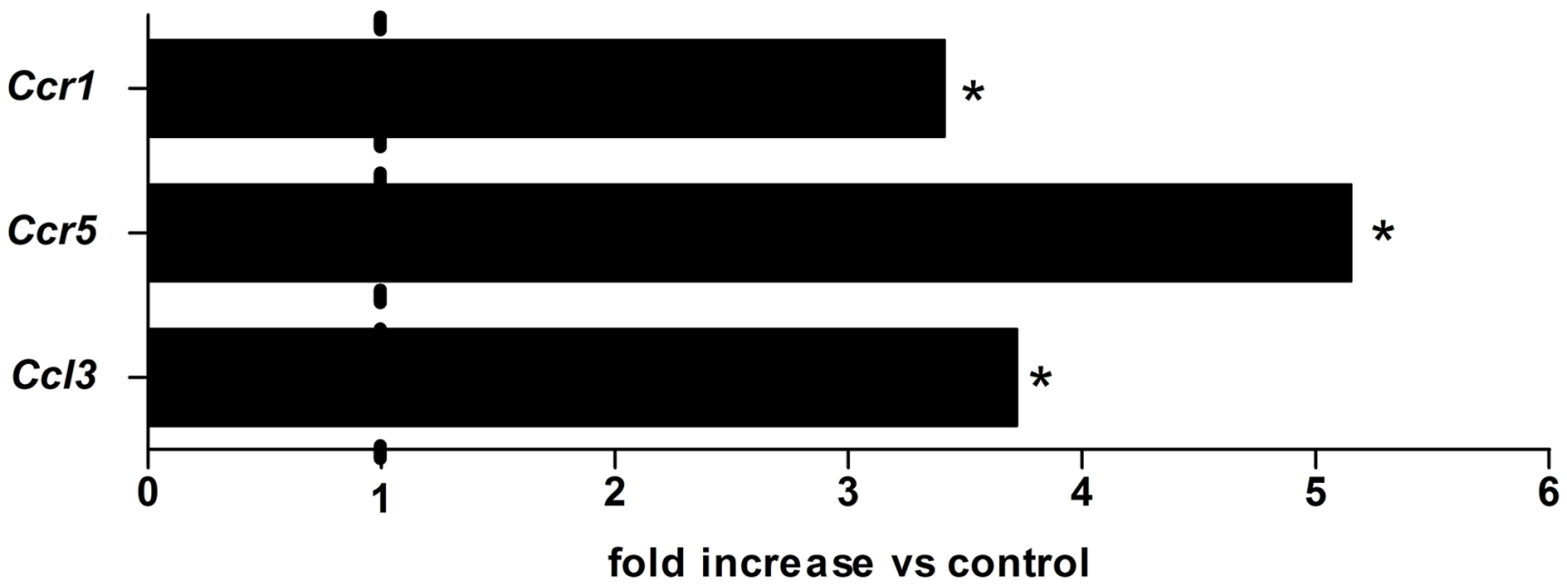
The expression of Ccl3, Ccr1 and Ccr5 in 4NQO-induced SCC lesions Mice were treated with the chemical carcinogen 4NQO during 28 weeks when lesions were obtained for qPCR analysis. Control mice received drinking water during the experimental period. n=5 per group. * p< 0.05 compared to the control.

The expression of Ccl3 (4.0 fold) and its receptors Ccr1 and Ccr5 (3.5 and 5.0 fold, respectively) was significantly increased in 4NQO-induced oral tumours (*p*<0.05) (Figure 1). CCL3 protein secretion was further confirmed by ELISA (108.7±37.5 and 348.6±181.3 pg/100 mg tissue, control and treated group, respectively) and similar results were achieved. Consistently, immunolocalization of CCL3 and CCR5 in tongue lesions showed positivity in neoplastic cells and stromal/inflammatory cells (Supplementary Figure 1).

CCR1 and CCR5 mRNA expression was also evaluated in HN12 and HN13 cell lines and results showed a significant increase of both receptors (CCR1 5.0 and 4.5 fold, and CCR5 3.5 and 4.0, respectively) in relation to human normal oral keratinocyte cell line (NOK). Fragments of human oral healthy mucosa and human healthy skin were also included in this analysis, but no expression of CCR1 and CCR5 was detected in these samples. Fragments of human inflamed oral mucosa and inflamed skin were used as positive controls and showed significant augment of CCR1 and CCR5 expression in comparison with non-inflamed tissues (CCR1 7.0 and 4.0 fold, and CCR5 6.0 and 6.0 fold, respectively). The CCL3 and CCR5 positivity was further confirmed in HN12 cell line by using flow cytometry. Results showed that most of HN12 cells (97.6%) expressed CCL3, while CCR5 expression was verified in 0.1% of cells (Supplementary Figure 2).

Next, we analysed CCL3 expression in human samples of healthy oral mucosa, potentially malignant disorder oral leukoplakia (OLK) with different grades of epithelial dysplasia, and primary OSCC samples. Cytoplasmic CCL3 expression was markedly up-regulated in the epithelial cells of OSCC (parenchyma) when compared with healthy oral mucosa and OLK (Supplementary Figure 3). Additionally, an increased CCL3 expression by epithelial cells of healthy oral mucosa, in relation to OLK, was observed. CCL3 was also detected in the connective tissue (stroma), and the results showed a significant increase in healthy oral mucosa compared to OLK with different grades of dysplasia. On the other hand, there was no difference between CCL3 expression in the stromal cells of the healthy oral mucosa and OSCC samples (Supplementary Figure 3). No significant correlations were found regarding CCL3 expression with OSCC clinical parameters (tumour size, presence of metastasis and location) (data not shown).

### SCC tumour formation is reduced in CCL3 and CCR5 deficient mice

**Figure 2:**
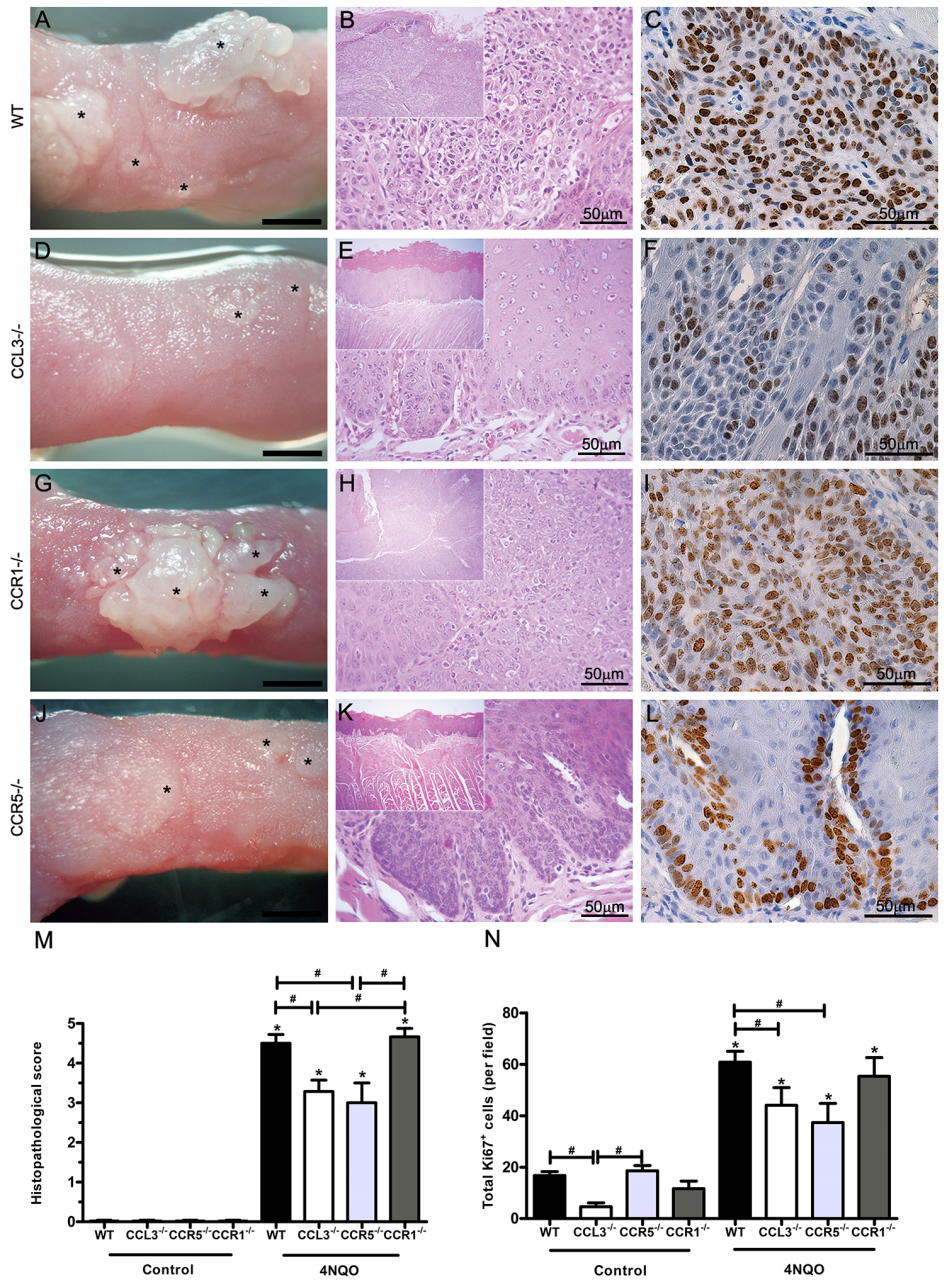
Clinical and microscopic findings of the SCC-induced lesions in WT, CCL3^-/-^, CCR1^-/-^ and CCR5^-/-^ mice after 4NQO treatment Macroscopic and microscopic appearance of SCC-induced lesions in WT (n=6) **(A, B)**, CCL3-/- (n=7) **(D, E)**, CCR1-/- (n=6) **(G, H)** and CCR5-/- (n=8) **(J, K)** mice. The asterisks indicate the clinical lesions (Bar = 0.1 cm). Ki67 immunoexpression in tongue lesions of WT **(C)**, CCL3-/- **(F)**, CCR1-/- **(I)** and CCR5-/- **(L)** treated mice. M and N. Histopathological scoring and quantification of the total Ki67^+^ cells in the experimental and control groups. *p<0.05 relative to the respective control, #p<0.05 when comparing groups of mice treated with 4NQO.

Our next approach involved inducing oral carcinogenesis in mice lacking CCL3 or its receptors CCR1 and CCR5. SCC was induced by 28 weeks of treatment with 4NQO, as previously reported [28, 29]. Clinically, SCC occurred as exophytic, papillomatous, white and base-attached tongue lesions (Figure 2A and 2G). Comparatively, the SCC tongue tumour formation was more prominent in the WT (Figure 2A) than the CCL3^-/-^ mice treated with 4NQO (Figure 2D). No changes in the tongue surface were observed in the control groups (data not shown). The microscopic analysis was consistent with the clinical findings and showed a pronounced cytomorphological atypia (*p*<0.05), with 100% of the lesions graded as carcinoma *in situ* and invasive carcinoma (scores 4 and 5, respectively) in the WT mice treated with 4NQO (Figure 2B and 2M). In contrast, CCL3^-/-^ mice treated with 4NQO presented lesions with a lower grade of cellular atypia and severity, with 57% of the lesions graded as moderate and severe dysplasia (scores of 2 and 3, respectively) (Figure 2E and 2M) and 43% classified as carcinoma *in situ*. Accordingly, the CCL3^-/-^ group treated with 4NQO had a significantly reduced Ki67 immunopositivity when compared with the WT mice (Figure 2F, 2C and 2N). No changes were observed in epithelium architecture in the control groups (Figure 2M).

CCR1^-/-^ mice treated with 4NQO presented a similar production of tumours (Figure 2G) and histopathological score (Figure 2H, 2M) in comparison with the WT mice (Figure 2M). In contrast, CCR5^-/-^ mice treated with 4NQO exhibited a reduced incidence of tumour formation (Figure 2J) and lower histopathological scores (Figure 2K and 2M), which was similar to the CCL3^-/-^ mice (Figure 2M). Moreover, the proliferative activity was decreased in the SCC lesions from CCR5^-/-^ mice treated with 4NQO compared with the CCR1^-/-^ mice (Figure 2L, 2I and 2N). The CCR1^-/-^ and CCR5^-/-^ control groups did not present any changes in tongue surface (data not shown) or abnormalities in epithelium architecture (Figure 2M).

To confirm the relative protection of CCL3 deletion in oral carcinogenesis, a more “aggressive” OSCC model using a high dose of 4NQO (200 µg/mL) was used [29]. The macroscopic and microscopic analyses revealed a similar incidence and clinical features of the SCC lesions in the tongue of the WT mice treated with 50 or 200 μg/mL of 4NQO. Again, the WT mice exhibited an increased tumour formation when compared with the CCL3^-/-^ mice. Noteworthy, while the WT mice exhibited pronounced cytological atypia and dysplasia (100% of lesions were graded as invasive carcinoma – score 5), the CCL3^-/-^ mice treated with a higher dose of 4NQO did not present any score of “invasive carcinoma” (data not shown). The histopathological analysis of the cervical lymph nodes and organs revealed no occurrence of metastasis after treatment with different doses of 4NQO. However, the liver of the treated mice presented variable degrees of hepatocytes tumefaction, steatosis and haemorrhage. The stomach and intestine also presented variable degrees of hyperqueratosis, hyperplasia and inflammation. These findings were similar for all of the groups of treated mice (data not shown).

### Decreased infiltration of eosinophils in SCC tumours in absence of CCL3

**Figure 3:**
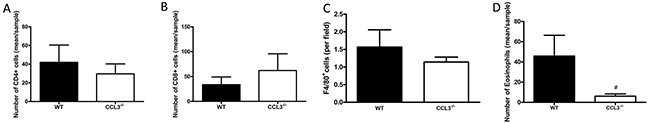
Inflammatory cell infiltrate in SCC-induced lesions Immunohistochemistry analysis of CD4^+^ **(A)** and CD8^+^ **(B)** lymphocytes and F4/80^+^ macrophages **(C)** in SCC lesions of WT and CCL3^-/-^ mice. Total number of Sirius Red stained eosinophils **(D)** in SCC-induced lesions of CCL3^-/-^ and WT mice. n=4-5 mice per group. #p<0.05 when compared to WT mice.

We next determined if CCL3 deletion modifies the inflammatory cell infiltrate in SCC lesions. Results showed that numbers of immunostained CD4^+^ and CD8^+^ lymphocytes and F4/80^+^ macrophage cells were similar comparing WT and CCL3^-/-^ treated groups (Figure 3A, 3B and 3C). In contrast, the number of infiltrating Sirius Red-stained eosinophils was remarkably reduced in tongue lesions of CCL3^-/-^ mice (Figure 3D).

### SCC lesions of CCL3 deficient mice have a reduced expression of angiogenic factors and ECM components

**Figure 4:**
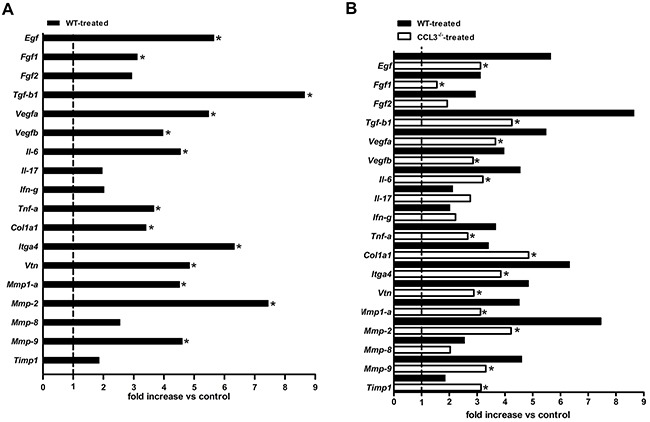
Expression of angiogenic, pro-inflammatory cytokines, adhesion molecules and extracellular matrix components in the SCC-induced lesions Molecular expression was determined by qPCR array in lesions from WT and CCL3^-/-^ mice. n=5 per group. Comparative analysis of WT control and 4NQO-treated mice **(A)**. Comparison between the WT and CCL3^-/-^ mice treated with 4NQO **(B)**. The results are expressed as the fold change relative to the control group after being normalized as the initial geometric mean of three constitutive genes (GAPDH, ACTB, and Hprt1). * p<0.05.

Because we have observed reduced SCC formation in the CCL3^-/-^ mice, we then evaluated whether the expression of angiogenic factors, cytokines and ECM components would be consistently diminished in the tumour milieu by the absence of CCL3. SCC lesions of WT mice treated with 4NQO presented a significantly increased expression of the proliferative and angiogenic factors *Egf*, *Fgf1, Tgfβ1, Vegfa* and *Vegfb* and the inflammatory cytokines *Il-6* and *Tnf-α*. Additionally, there was an increased expression of the matrix components *Col1a1*, *Mmp-1a*, *Mmp-2*, *Mmp-9* and the adhesion molecules *Itga4* and *Vtn* in the WT mice treated with 4NQO in relation to the WT control group (Figure 4A). On the other hand, the CCL3^-/-^ mice treated with 4NQO had a significantly decreased expression of the angiogenic factors *Egf*, *Fgf1*, *Tgfβ1*, *Vegfa*, and *Vegfb*, the inflammatory cytokines *Il-6* and *Tnf-α*, the matrix components *Mmp-1a*, *Mmp-2*, and *Mmp-9*, and the adhesion molecules *Itga4* and *Vtn* (Figure 4B).

### CCL3 induced the expression of cytokines and MMPs by neoplastic cells

**Figure 5:**
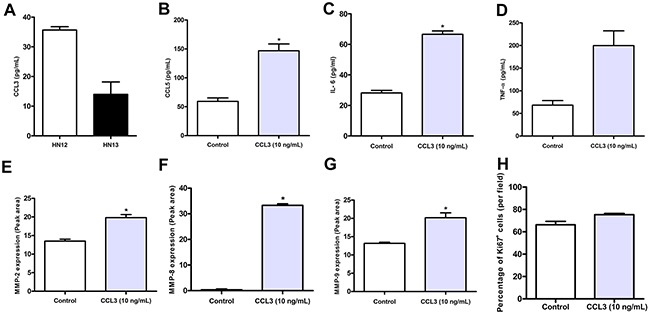
Comparative production of CCL3 by metastatic HN12 and non-metastatic HN13 tumour cell lines CCL3 **(A)** treatment induces the release of CCL5 **(B)**, IL-6 **(C)**, TNF-α **(D)**, MMP-2 **(E)**, MMP-8 **(F)** and MMP-9 **(G)** by HN12 cells. Percentage of the total HN12 Ki67^+^ cells stimulated with CCL3 **(H)**. Cells were stimulated or not (control) with CCL3 (10 ng/mL) as described in Material and Methods. The experiments were performed in triplicate and the results are representative of three independent experiments. The means ± SD were calculated and are as shown. *p<0.05 relative to control.

The evidence obtained in our previous experiments suggested that a lack of CCL3 turned down pathways associated with invasiveness and proliferation in SCC. Next, we sought to investigate whether CCL3 directly stimulates neoplastic cells. Initially, we assessed CCL3 production by HNSCC metastatic (HN12) and non-metastatic (HN13) cell lines under steady state conditions and observed that the metastatic lineage produced increased levels of CCL3 in relation to the non-metastatic (35.69±1.55 and 13.97±8.35, respectively) (Figure 5A). HN12 was then selected for the subsequent experiment due to its metastatic behaviour. We then determined the effect of different CCL3 concentrations (5, 10 or 20 ng/mL) on cellular viability. CCL3 at 10 ng/mL did not disrupt cell integrity, and this concentration was used in the subsequent experiments. The results showed that under CCL3 stimuli, HN12 cells produced significantly increased levels of CCL5 and IL-6 compared with the control (Figure 5B and 5C). The levels of TNF-α did not increase significantly after CCL3 stimulation (Figure 5D). Extracellular matrix component degradation is an essential step in tumour invasion [30], and therefore, we analysed whether CCL3 induces the expression of matrix metalloproteinases (MMPs). Indeed, there was a significant increase in the production of MMP-2, MMP-8 and MMP-9 after CCL3 treatment (Figure 5E, 5F and 5G). On the other hand, HN12 cultures stimulated with CCL3 had similar proliferation levels, as measured by the expression of Ki67, in comparison with the control (non-stimulated HN12 cells in DMEM-serum free media) (Figure 5H).

### Blockade of CCL3 significantly impaired the invasion of HN12 cells *in vitro*

**Figure 6:**
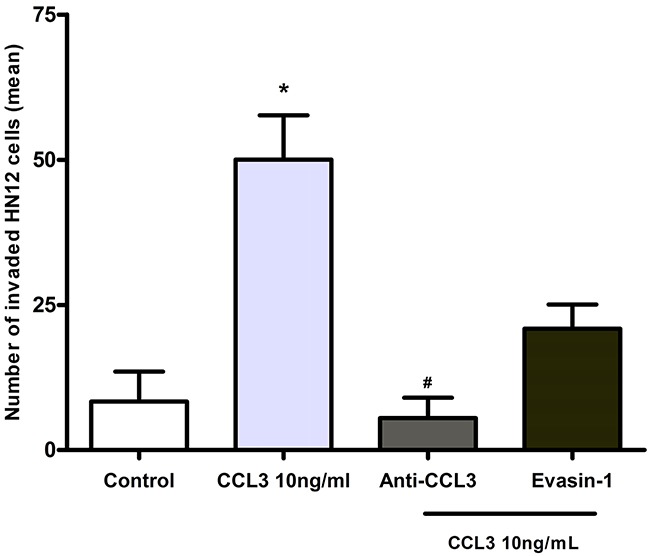
Effects of CCL3 blockade in the invasion of HN12 cells The cells were first pre-treated with an anti-CCL3 antibody (20 µg/mL) or Evasin-1 (10-7 M) and then were stimulated with CCL3 (10 ng/mL). The non-stimulated HN12 cells comprised the control group. The Y-axis represents the quantification of the total number of invading HN12 cells. The experiments were performed in triplicate, and the results from three independent experiments were considered (means ± SD). *p<0.05 relative to control. #p<0.05 when compared with the group stimulated with CCL3.

Previous results showed that CCL3 stimulates release of enzymes involved in ECM remodelling by HN12 cells. Then, we determined whether this effect would result in augment of neoplastic cell invasion *in vitro*. The results showed that after treatment with CCL3 (10 ng/mL), a significant increase of cells that invaded through membranes was seen in comparison with the control group (*p*<0.05; Figure 6).

**Figure 7:**
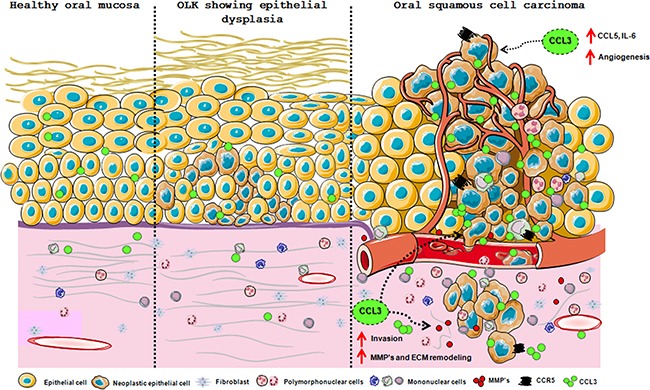
Schematic presentation of the CCL3/CCR5 axis involvement in oral carcinogenesis Oral healthy mucosa and potentially malignant lesion OLK exhibit low expression of CCL3 in epithelium and connective tissue. During neoplastic epithelial transformation this expression is increased and persists in the established lesions. CCL3 released by neoplastic cells and immune infiltrated cells creates a pro-tumorigenic microenvironment by inducing the secretion of chemokines and cytokines, inflammatory cell recruitment, e.g. eosinophils, angiogenesis and degradation of extracellular matrix. These CCL3 functions are exerted via CCR5 expressed in inflammatory and neoplastic cells. Overall, CCL3/CCR5 actions culminate in neoplastic cell invasive phenotype.

We further analysed whether therapies blocking CCL3 could prevent invasion. Treatment with an anti-CCL3 antibody significantly impaired the invasion of HN12 cells in relation to cells treated with CCL3. Evasin-1 also decreased the number of invading cells (Figure 6). Our results provide definitive evidence that CCL3 is relevant for oral carcinogenesis, regulating tumour cell interplay and neoplastic cell behaviour. The schematic Figure 7 summarizes the hypothesis of CCL3/CCR5 functions in oral carcinogenesis (Figure 7).

## DISCUSSION

The main findings of this study can be summarized as follows: 1) Experimentally induced SCC tumours exhibit enhanced expression of CCL3 and its receptors CCR1 and CCR5; 2) The absence of CCL3 and CCR5, but not CCR1, reduced chemically induced SCC formation, as confirmed by clinical presentation, decreased histopathological scores and lower proliferative activity of lesions; 3) Oral carcinogenesis, in the absence of CCL3 is associated with decreased eosinophils infiltration and attenuated expression of angiogenic factors, cytokines and matrix metalloproteinases; 4) *In vitro*, CCL3 directly stimulate tumour cells to invade and produce cytokines and matrix metalloproteinases; 5) The specific effects of CCL3 on tumour cells were proved by using CCL3 inhibitors. Finally, our results clearly demonstrated the relevance of CCL3 acting via CCR5 in oral carcinogenesis. This is the first study to provide mechanistic data linking CCL3/CCR5 to oral cancer.

We first observed an increased mRNA expression of Ccl3 and Ccr5 in experimentally-induced tongue carcinomas. Then, we confirmed that both stromal and neoplastic cells are positive cells for CCL3 and CCR5. Consistently, oral carcinoma cell lines also express CCL3 and CCR5. Accordingly, CCL3 levels were increased in different types of tumours as hepatomas [19], multiple myeloma [24] and chronic lymphocytic leukaemia [25]. We also verified the contribution of epithelial cells expressing CCL3 in human OSCC samples, which was higher compared with healthy oral mucosa and the potentially malignant disorder OLK. OLK with different grades of epithelial dysplasia exhibited similar CCL3 expression, which may suggest that this chemokine does not influence the early stages of oral carcinogenesis. However, this topic needs to be further explored because malignant transformation rates in OLK could not be clearly defined [31]. In OSCC, CCL3 is significantly expressed in the epithelium (by neoplastic cells/parenchyma) while in healthy oral mucosa, there is a balanced expression in the epithelium and connective tissue. These findings might also account for the role of CCL3 in oral mucosa homeostasis, as described in other processes [32].

Previous data from our group showed that CCL3 expression in primary OSCC was related with poor cumulative survival rates [27]. These results indicate a possible functional role for CCL3 in oral tumorigenesis and prompted us to explore it. Because the intrinsic limitations, due to the variability of tumours, stage and parameters analysed in studies of human samples we herein used a model of oral carcinogenesis by the administration of the water-soluble chemical carcinogen 4NQO. In our conditions, 4NQO treatment was efficient to produce tongue tumours as described [28, 29, 33] resulting in the formation of squamous cell carcinoma lesions in mouse tongue. In the absence of CCL3 and CCR5, but not of CCR1, SCC formation was significantly reduced. Accordingly, the microscopic analysis revealed decreased histopathological scores and a decreased proliferative activity of the lesions in mice lacking CCL3/CCR5. These morphological data demonstrated that CCL3/CCR5 system is critical for oral carcinogenesis, specifically in the initiation and promotion steps. However, the participation of CCL3/CCR5 axis in established tumour could not be clearly defined. It is a limitation of this study because the long-time treatment with 4NQO for producing tumours and consequently its toxicity precludes its use as a model to analyse tumour progression or response to treatments. Similarly to our findings, previous studies found that deficiency of the CCL3/CCR5 system resulted in a significantly reduced tumour formation and a reduced lung metastasis [21, 22]. On the other hand, a study demonstrated that the absence of CCL3/CCR1, but not CCL3/CCR5, resulted in a decreased incidence of hepatocellular carcinoma [20]. The differences in the mouse models and the tumours in these studies could explain such a discrepancy.

The participation of chemokines and receptors in OSCC development was previously suggested [13–18, 27]. Herein, the increase of Ccl3, Ccr1 and Ccr5 in SCC mouse tumours is indicative of contribution of these molecules to oral carcinogenesis. Primary effect of chemokines in homeostatic conditions [32] and pathological conditions e.g. cancer [34] is to induce the inflammatory cell trafficking. Unexpectedly, CCL3 deficiency did not influence recruitment of macrophages or lymphocytes to SCC lesions. In contrast, we observed reduced intratumoral infiltration of eosinophils in CCL3 deficient mice. In line with these findings, eosinophil depletion protects mice from 4NQO-induced oral carcinogenesis [33]. In addition, tumour-associated eosinophilia (TATE) was associated with progression of oral cancer [35]. Thus, the recruitment of eosinophils would contribute to the observed phenotype in CCL3 deficient mice, a tenet that deserves further investigation.

In attempt to dissect whether CCL3 influences the tumour milieu, we evaluated the expression of molecules involved in tumour proliferation (EGF, FGF, TGF-β and TNF-α) [36, 37] and angiogenesis (TGF-β1, VEGFa and VEGFb) [38–40]. We demonstrated that in the absence of CCL3, the expression of markers *Egf*, *Fgf1*, *Tgf-β1*, *Vegfa*, V*egfb*, *Il-6*, *Tnf-α*, *Itga4* and *Vtn* was significantly reduced. Our results are corroborated by previous findings showing that tumours from CCL3^-/-^ and CCR5^-/-^ mice presented reduced vascularisation [21]. The decreased expression of the adhesion molecules *Itga4* and *Vtn* in the SCC lesions from the CCL3^-/-^ mice may be indicative of the fact that CCL3 activate neoplastic cell adhesion and motility within the tumour microenvironment as described for other tumours [41]. Furthermore, we found a decreased production of cytokines IL-6 and TNF-α in the 4NQO-induced lesions of the CCL3^-/-^ mice. In line with these findings, blockade of the IL-6 receptor reduces tumour growth and angiogenesis *in vivo* [42], and the expression of IL-6 and TNF-α in OSCC cell lines correlated with the metastatic phenotype [43, 44]. Therefore, changes in inflammatory and angiogenic factors appear to be the underlying mechanism explaining the OSCC phenotype in CCL3 deficient mice.

*In vitro* experiments account for a direct effect of CCL3 in neoplastic cells. In this study, two different HNSCC cell lines were used to assess these effects – HN12 (which presents a metastatic behaviour) and HN13 (non-metastatic). Under steady state conditions, the HN12 cells produce significant CCL3 levels *in vitro*, as previously reported [45]. Noteworthy, HN12 cells produce CCL5, IL-6 and TNF-α under CCL3 stimuli *in vitro*. Consistently, the up-regulation of these was also observed in mouse tumours in this study. The invasion of tumour cells into adjacent tissues requires tumour microenvironment remodelling, which is a critical step for metastasis [30]. In this regard, the production of MMPs in OSCC tissues is key in this process [46, 47]. *In vitro*, we verified that HN12 cells showed an increased MMPs production and invasiveness when stimulated with CCL3. Interesting, the SCC samples from the CCL3 deficient mice exhibited a diminished expression of *Mmp-1a*, *Mmp-2* and *Mmp-9*. Additionally, we observed that *Timp1* and *Col1a1* were consistently up-regulated in the absence of CCL3. These results are similar to previous findings pointing out that CCL3 is a stimuli for MMPs expression in metastatic murine lung tumours [21].

Confirming the specificity of the CCL3 effect, a significant reduction in cellular invasion was observed by CCL3 blockage with Evasin-1, a CCL3 binding protein [48–50]. Consistently, previous data showed that CCL3 induced pseudopodia formation in hepatoma cells [26]. Therefore, the use of specific drugs to inhibit CCL3/CCR5 activity could represent potential strategies for oral cancer.

Current findings provide definitive evidence of the contribution of the CCL3-CCR5 axis in experimental oral carcinogenesis. A proposal model of the CCL3/CCR5 functions in oral carcinogenesis is presented in Figure 7. Oral healthy mucosa and potentially malignant lesion OLK exhibit low expression of CCL3 in epithelium and connective tissue. During neoplastic epithelial transformation this expression is increased and persists in the established lesions. CCL3 released by neoplastic cells and immune infiltrated cells creates a pro-tumorigenic microenvironment by inducing the secretion of chemokines and cytokines, inflammatory cell recruitment, e.g. eosinophils, angiogenesis and degradation of extracellular matrix. These CCL3 functions are exerted via CCR5 expressed in inflammatory and neoplastic cells. Overall, CCL3/CCR5 actions culminate in neoplastic cell invasive phenotype.

## MATERIALS AND METHODS

### Animals

C57BL/6 (wild type - WT) 6-8-week-old male mice were obtained from Centro de Bioterismo, Instituto de Ciências Biológicas (CEBIO), Universidade Federal de Minas Gerais, Brazil. CCL3^-/-^ mice were bred by and obtained from Centro de Pesquisas René Rachou (Fiocruz Minas Gerais, Brazil). CCR5^-/-^ mice were generated as previously described [51], and CCR1^-/-^ mice were obtained from Taconic Farms (Hudson, New York, USA). The mice were maintained under standard conditions with a 12 h light/dark cycle, a controlled temperature (24 ± 2 °C) and free access to commercial chow and water *ad libitum*, according to the Ethics Committee in Animal Experimentation (protocol 12/2011).

### OSCC induction

The induction of oral carcinogenesis was performed as previously described [29, 33]. Briefly, 4-nitroquinoline-1-oxide (4NQO) (Sigma-Aldrich, St. Louis, MO, USA) was dissolved in ethylene glycol (Sigma-Aldrich) at 50 µg/mL or 200 µg/mL and was stored at 4°C. Experimental mice received 4NQO daily for 28 weeks, and the control mice received drinking water only. After 28 weeks, the mice were euthanized and the tongue, cervical lymph nodes, liver, lungs, stomach, duodenum, jejunum, ileum and large intestine were collected for microscopic analysis.

### Histopathological analysis

The organs, including the cervical lymph nodes, liver, lungs, stomach, duodenum, jejunum, ileum and large intestine, and also tongues from WT, CCL3^-/-^, CCR1^-/-^ and CCR5^-/-^ mice treated with 4NQO (n=6-8 per group) were fixed in 10% buffered formalin, embedded in paraffin wax, longitudinally cut (3 µm sections) and stained with H&E. The tongue tumours were classified using the following adapted score [52]: 0 – normal epithelial architecture; 1 - mild dysplasia (changes limited at basal third of lining epithelium); 2 - moderate (changes in two-thirds of lining epithelium); 3 - severe (more than two-thirds); 4 - carcinoma *in situ* (full thickness of the lining epithelium, without invasion of connective tissue) and 5 - invasive carcinoma (carcinomatous islands into connective tissue). Twenty consecutive fields were evaluated by two examiners (J.M.S and T.A.S) blinded to the group status. The organs were carefully analysed by a general pathologist (M.A.R) to assess the possible effects of 4NQO intake at these distant sites. The Intraclass Correlation Coefficient test was performed (ICC = 0.83) to validate the reliability of the inter- and intra-examiner evaluations.

### Immunohistochemistry and cell counting

Immunohistochemistry was performed on the tongue sections using the streptavidin-biotin method [33]. Briefly, the slides were deparaffinised, dehydrated and rinsed in distilled water, followed by incubation with 0.3% hydrogen peroxide and Avidin/Biotin blocking system (Dako, Carpinteria, CA, USA). The sections were then incubated with a monoclonal mouse anti-human Ki-67 (MM1, Novocastra, Newcastle, UK, 1:50), polyclonal goat IgG anti-mouse CCL3 (450-MA, R&D Systems, Minneapolis, MN, USA, 1:100), mouse monoclonal anti-CCR5 (D-6, Santa Cruz, Dallas, TX, USA, 1:100), the rat anti-mouse F4/80 (RM2900, Caltag, Buckingham, MK, UK at 1:100), anti-mouse CD4 (GK1.5, eBioscience, San Diego, CA, USA, 1:100) and anti-mouse CD8a (4SM15, eBioscience, San Diego, CA, USA, 1:100), 4 °C overnight. Negative controls were obtained by omission of the primary antibody, which was substituted with 1% PBS-BSA. The cells were analysed by a light microscope (Axioskop 40 Zeiss, Carl Zeiss, Gottingen, Germany) at 1000x original magnification and counted in 20 consecutive fields in two sections.

### Sirius Red staining

Sirius Red staining is a method used to access the presence of eosinophils [33]. Briefly, slides (n=5 per group) were incubated in Harris hematoxylin (two minutes), rinsed in tap water and in 100% ethanol. Subsequently, slides were immersed in an alkaline (pH 8–9) Sirius Red solution (CI 35780, Sigma Aldrich) during two hours. Stained eosinophils were counted in 20 consecutive fields of the epithelium lining area (including a third of lamina propria, under epithelial layer), at 400x magnification. Results were expressed as a total number of eosinophils per sample.

### Human samples analysis

Thirty-three cases of OSCC, 39 cases of potentially malignant oral disorder diagnosed as oral leucoplakia (OLK) and 16 cases of clinically healthy oral mucosa (controls) were obtained from the Laboratório de Patologia Bucal, Faculdade de Odontologia, Universidade Federal de Goiás and Hospital Araújo Jorge, Associação do Combate ao Câncer em Goiás, Goiânia (Ethics Committee approval 013/2010). The OLK samples were graded as described [52] for epithelial dysplasia (20 light, 14 moderate and 5 high). CCL3 expression was evaluated by immunohistochemistry as described above using a rabbit polyclonal anti-human CCL3 (FL-92, Santa Cruz, Dallas, TX, USA) at 1:50. CCL3 expression was quantified in the epithelium and connective tissue by light microscopy in 10 consecutive fields at 400x original magnification.

### Enzyme-linked immunosorbent assay

For ELISA, the SCC tongue lesions and the clinically normal tongue samples (n=3 per group) from WT mice were weighed and homogenized as described previously [33]. The homogenate was centrifuged (8,946 x g) at 4 °C for 10 min, and the supernatant was stored at -70 °C until the analysis. The concentration of CCL3 was measured using a commercially available kit (R&D Systems, Minneapolis, MN, USA). The results are expressed as the mean of picograms of cytokines normalized to 100 mg of tissue ± standard deviation (SD).

### Real time PCR array

A Real Time PCR array was performed as previously described [53]. Total RNA from SCC-induced tongue lesions; the human cell lines HN12, HN13 and normal oral keratinocyte (NOK); human samples of oral healthy mucosa, healthy skin, inflamed oral mucosa and inflamed skin (used as controls) was obtained with the RNeasyFFPE kit (Qiagen Inc, Valencia, CA, USA) according to the manufacturer's instructions. A Real Time PCR array was performed in a Viia7 instrument (LifeTechnologies, Carlsbad, CA, USA) using the custom panels “Wound Healing” (PAMM-121) and “Inflammatory cytokines and receptors” (PAMM-011) (SABiosciences, Frederick, MD, USA). The data were analysed by the RT2 profiler PCR Array Data Analysis online software (SABiosciences) for normalizing the initial geometric mean of three constitutive genes (*GAPDH*, *ACTB* and *Hprt1*), normalized by the control group and expressed as the fold change relative to the control group.

### Cell culture and MTS assay

The head and neck squamous cell carcinoma (HNSCC) cell lines (HN12 – metastatic obtained from metastatic lymph node and HN13 – non metastatic from primary squamous cell carcinoma of the tongue) [54] were cultured in Dulbecco's modified medium - DMEM (Sigma-Aldrich), supplemented with 10% FBS (Gibco, Carlsbad, CA, USA), antibiotics and antimycotics (Cat. A5955, Sigma-Aldrich) in 5% CO^2^ at 37 °C. The CellTiter 96® AQueous One Solution Cell Proliferation Assay (Promega, Madison, WI, USA) was used to determine the cell viability according to the manufacturer's instructions. The cells (1×10^5^) were plated on 96-well plates (Corning Inc., Corning, NY, USA) for 24, 48 and 72 hours before the addition of the MTS solution (a tetrazolium compound and phenazine methosulfate) (Promega).

The culture supernatants were collected at 24, 48 and 72 hours for ELISA using commercially available kits for the detection of CCL3, CCL5, IL-6 and TNF-α (R&D Systems) as previously described.

### Flow cytometry

1×10^6^ HN12 cells were first incubated with Brefeldin-A 1 mg/mL (Sigma) at 37° C, 5% CO_2_ during 3 hours. Subsequently, cells were rinsed with PBS and incubated with antibodies PE mouse anti-human CD195 (CCR5) (Cat. 556042, BD Pharmingen, at 1:20 dilution) or isotype control mouse IgG2a (Cat. 550339, BD Pharmingen, at 1:100) during 30 minutes. Intracellular staining was performed by using the rabbit polyclonal anti-human CCL3 (clone FL-92, Santa Cruz, at 1:50) conjugated with anti-rabbit IgG (H+L) Alexa Fluor 647 (Cat. 4414, Cell Signaling, at 1:50) during 30 minutes each step. Cells were analyzed with a FACScalibur CantoII, and data were analyzed by FlowJo (Treestar, Ashland, OR, USA).

### Proliferation assay

The cells (1×10^5^) were plated in 24-well plates (Corning Inc.) for 48 hours in serum-free DMEM (control) or in the presence of CCL3 (10 ng/mL). After incubation, the supernatant was removed and immunocytochemistry was performed as described above using an anti-Ki67 antibody (ab15580, Abcam, Cambridge, England). Ki67 positive cells were counted using Image J software.

### Zymogram assay

The cells (3×10^5^) were plated in a 6-well plate. After 24 h, the medium was changed to 1 ml of serum-free DMEM, and then, the cells were treated or not (control) with CCL3 (10 ng/mL) and maintained for an additional 24 and 72 hours. Twenty microliters of the supernatant were resolved by 12% SDS-PAGE (Cat. SM1841, Fermentas, Kent, UK) containing 1 mg/mL gelatin. The gel was washed with 2% Triton X-100, incubated in 10 mM Tris–HCl and 5 mM CaCl2 for 16 hours at 37 °C and, then, was stained with 0.25% Coomassie blue (Sigma-Aldrich).

### Invasion assay

The cell invasion assay was performed in 24-well plates using modified Boyden chamber inserts with a polycarbonate filter membrane containing 8 μm pores. Matrigel (Cat.35423C, BD Biosciences, Bedford, MA) was diluted 1:1 with a serum-free medium and used to coat the filter membranes. The cells (2×10^5^) were pre-treated with CCL3 (Cat. DY270, R&D Systems) (10 ng/mL), antibody anti-CCL3 (FL-92; Santa Cruz Biotechnology) (20 µg/mL) or Evasin-1 (AS9001965, 10^-7^ M) (Serono Pharmaceutical Research Institute SA, Geneva, Switzerland), a chemokine-bind protein that depletes CCL3 [50]. After 45 minutes, the cells were re-suspended in 250 μL of serum-free DMEM and seeded onto the upper compartment. DMEM containing CCL3 (10 ng/mL) was used in the lower chamber for stimulation. After 72 h, the filters were fixed in 10% formalin for 15 min. The cells on the lower surface were stained with Giemsa (Sigma-Aldrich). Five fields were photographed at 200x original magnification using a Zeiss Axiovert 40 inverted microscope and were processed using the AxioVision Rel. 4.8.2 software (Carl Zeiss).

### Statistical analyses

The statistical analyses were performed using the software GraphPad Prism 5.0 (GraphPad Software Inc., San Diego, CA, USA). A Student's t test was performed after checking for data normality. A comparative analysis was performed by the Kruskall-Wallis, followed by Dunn's multiple comparison post test for human specimens. Correlations between CCL3 expression in parenchyma and stroma and clinical parameters of human OSCC tumours were accessed using *Spearman’s* correlation coefficients and/ or *chi-square Pearson* tests. The results are reported as the means ± SD. *p* values <0.05 are considered statistically significant.

## SUPPLEMENTARY MATERIALS FIGURES



## Abbreviations

CCL3: CC chemokine ligand 3
CCR5: CC chemokine receptor 5
CCR1: CC chemokine receptor 1
OSCC: Oral squamous cell carcinoma
WT: Wild type
4NQO: 4-Nitroquinoline-1-oxide
OLK: Oral leucoplakia
HNSCC: Head and neck squamous cell carcinoma
MMPs: Matrix metalloproteinases
SCC: Squamous cell carcinoma
ECM: Extracellular matrix
