# Supplementary material for: Relevance of CCL3/CCR5 axis in oral carcinogenesis
Source: Oncotarget. 2017 Apr 6;8(31):51024–36. doi: 10.18632/oncotarget.16882 (PMC5584227; doi:10.18632/oncotarget.16882)
Supplement: Supplementary file 1 [file oncotarget-08-51024-s001.pdf]

Relevance of CCL3/CCR5 axis in oral carcinogenesis

SUPPLEMENTARY FIGURES

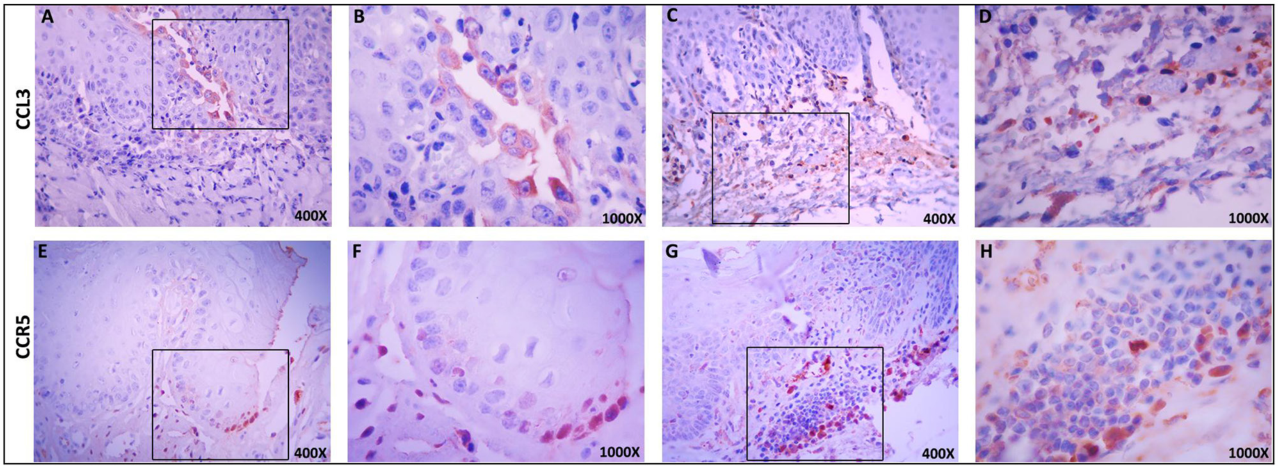

Supplementary Figure 1: CCL3 and CCR5 expression in chemically induced lesions. CCL3 positivity in parenchyma **(A and B)** and stromal cells **(C and D)**. CCR5 immunoexpression by tumour cells **(E and F)** and stromal cells **(G and H)**. n=4 per group. Areas delimited by squares in **(A), (C), (E)** and **(G)** (400x) are showed in higher magnification in **(B), (D), (F)** and **(H)** (1000x original magnification), respectively.

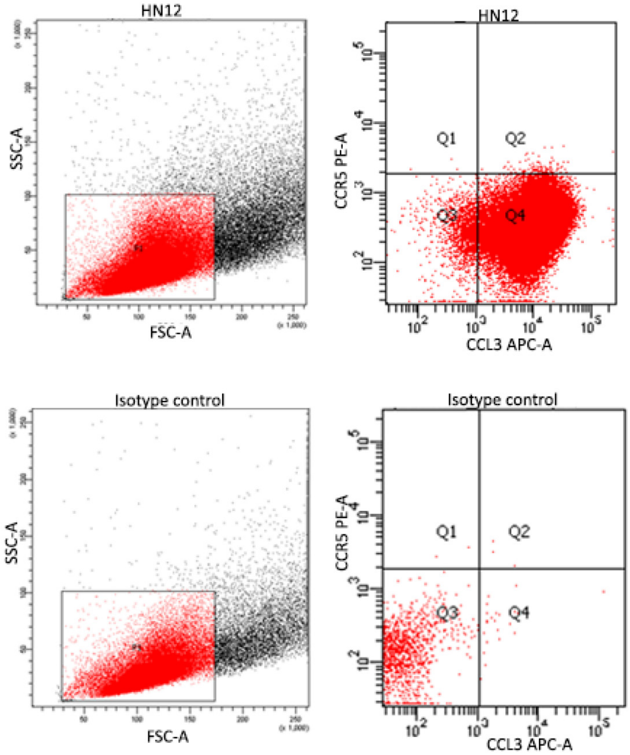

Supplementary Figure 2: CCL3 expression by the metastatic tumour cell line HN12. 1x10^6 cells were incubated with anti-CCL3-APC and anti-CCR5-PE antibodies and analyzed by flow cytometry. Top and bottom panels represent the HN12 CCL3/CCR5 expression and the isotype-matched control, respectively.

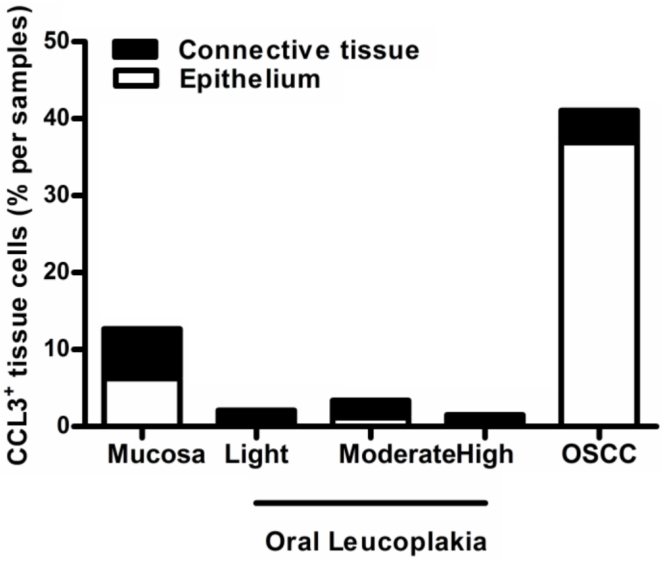

Supplementary Figure 3: CCL3 expression in oral mucosa, oral leucoplakia (OLK) and OSCC. The expression of CCL3 was determined in human samples of healthy mucosa, OLK with different grades of epithelial dysplasia and OSCC primary tumours using immunohistochemistry. The results are presented as the mean percentage of $CCL3^+$ cells in epithelium (parenchyma) and connective tissue (stroma). Comparative analyses were performed by the Kruskall-Wallis, followed by Dunn's multiple comparison post test.
